# The Construction of Positive Social Psychology During the COVID-19 Pandemic: Exploring the Relationship Between Social Support and Peace of Mind Through a Three-Wave Longitudinal Cross-Lag Study

**DOI:** 10.3389/fpsyg.2021.631748

**Published:** 2021-10-26

**Authors:** Yiheng Xi, Li Zhou, Ying Wang

**Affiliations:** ^1^Department of Labor and Human Relations, Renmin University of China, Beijing, China; ^2^Department of Political Science, Faculty of Social Sciences and Solvay Business School, Vrije Universiteit Brussel, Brussels, Belgium; ^3^Department of Linguistics and Literary Studies, Vrije Universiteit Brussel, Brussels, Belgium; ^4^Department of Economics and Management, Nanjing University of Aeronautics and Astronautics, Nanjing, China

**Keywords:** the COVID-19 pandemic, Chinese sense of well-being, peace of mind, social support, cross-lag model

## Abstract

The ongoing COVID-19 pandemic has sparked a major global crisis that has infected public social mentality. Drawing on the concept of peace of mind (PoM), a culture-specific positive emotion construct developed in the Chinese cultural context, this study explored the ways to build a positive public social mentality in the time of the pandemic. PoM is indicative of a calm and stable emotional state marked by self-control and spiritual cultivation and is believed to align with the perceptions of subjective well-being in Chinese or eastern cultures. A three-wave cross-lag study using an online questionnaire survey was conducted on 107 employees in Chinese enterprises during the pandemic. The research findings suggest that social support had a significant positive time-cross effect on later PoM, i.e., social support-T1 had a significant predictive effect on PoM-T2 (β = 0.16, SE = 0.09, *p* < 0.05) and social support-T2 had a significant predictive effect on PoM-T3 (β = 0.38, SE = 0.19, *p* < 0.05), whereas PoM failed to show a positive time-cross effect on later social support, i.e., the predictive effects of PoM-T1 on social support-T2 (β = 0.04, SE = 0.07, *p* > 0.05) and of PoM-T2 on social support-T3 (β = 0.13, SE = 0.09, *p* > 0.05) were not significant. This study provided a dynamic picture of the construction of public social mentality in the time of public health emergencies and also contributed to the research on PoM antecedents.

## Introduction

The outbreak of the COVID-19 pandemic has posed a serious challenge to public health, both physically and psychologically. As countries around the globe are pressed for time in containing the pandemic, measures such as quarantine and social distancing in response to the pandemic have turned the life of the public upside down, resulting in a decline in the sense of control of people over their lives and even their safety. Additionally, the uncertainty and unpredictability of the social, economic, and international environment and even living conditions, as well as severe limitations on autonomous behavior and vague prospects, have jointly failed the public in perceiving the original public order promptly and clearly, which deteriorated into a variety of negative social emotions, such as group panic, anxiety, and irritability. This has greatly undermined the stability and positivity of both individual and social mentality (Dobrowolska et al., 2020). The social mentality in the time of the pandemic differentiates itself from that under normal circumstances. One of the most distinguishing features is that the pandemic-inflicted social mentality is more noticeable and changeable with the development of the pandemic due to its urgency and a high degree of risk (Qin et al., 2020). In this regard, an unstable mentality has become a manifestation of public social psychology. Therefore, it is critical to construct a positive social mentality in the prevention and control of the pandemic.

The social mentality is derived from the mental state of individuals and formed on the basis of psychological interaction, emotional fusion, and the interplay between individuals (Yang, 2006). As a result of the outbreak of the COVID-19 pandemic, the personal psychology of individuals has generally been in a state of stress and fear, and accordingly, the social mentality changed continuously. Some studies suggested that most people do not suffer from severe mental health problems caused by the pandemic (Chang et al., 2020; Zhong et al., 2020). However, the analysis of a variety of communities indicated that negative emotions such as panic are prevailing in the public at the time of the pandemic (Wang J. et al., 2020). Women reported more emotional experiences of fear than men during the COVID-19 pandemic (Broche-Perez et al., 2020; Liu et al., 2020; Rossi et al., 2020; Wang C. et al., 2020). By contrast, positive emotions, such as peace of mind (PoM), were rarely discussed.

Peace of mind is an emotional construct developed by Lee et al. (2013) to describe the distinctive affective well-being valued within the Chinese cultural context. It refers to an affective state involving inner peace and harmony, which has cultural and philosophical depth in Chinese society, as is evident from the basic principle of equilibrium (*zhong*) proposed by Confucianism and the balance between *Yin* and *Yang* (opposite forces) by Taoism. PoM is indicative of a stable emotional state marked by self-control and spiritual cultivation and claimed to be an ideal positive state for individuals sensitive to Chinese or eastern cultures (Lee et al., 2013). Lu and Gilmour (2004) indicated that “harmony” indicates a state of balance both within the mind of an individual as it emphasizes self-control and emotional regulation in approaching well-being and between an individual and his or her surroundings because it cultivates a socially conscious self, which is considerate to the needs of others and the community. The Chinese conception of well-being is, thus, perceived as “a dynamic process of achieving and maintaining a good fit from within and outward” (Lu and Gilmour, 2004, p. 286). A high level of PoM testifies to improved individual well-being and positive social mentality, which are of particular importance in times of public crisis.

However, the breakout of major emergencies, such as the COVID-19 pandemic, makes it difficult for individuals to maintain a peaceful and balanced mind, as a result, the social mentality is impaired (Shi, 2003), as is evident from the frequent occurrence of conflicts and panic buying during the pandemic. This may be attributed to the fact that people feel upset, isolated, and helpless in the face of the pandemic and that it becomes more difficult for them to gain a sense of control over themselves and the outside world. At a time when the certainty in the outside world and the normal order of life are suddenly disrupted following emergencies, the sense of control of the people is under serious threat. Therefore, the fact that people find it difficult to maintain a peaceful mind may be attributed to a sense of isolation and helplessness. Stay-at-home orders can be highly effective in terms of maintaining a physical distance. However, these measures may lead to loneliness and social isolation, especially in vulnerable groups (Aleman and Sommer, 2020; Benke et al., 2020; Brooks et al., 2020; Holmes et al., 2020), thereby impairing the state of PoM.

The sudden critical emergency of the COVID-19 pandemic is beyond the control of the public. However, keeping the public informed that the world is still orderly, regular, and certain is an efficient way to prevent the negative social mentality from continuing to spread, because it can serve as psychological compensation to make people feel that the living environment is still safe. Social support is an important way to form these perceptions. Social support refers to emotional or instrumental aid from others, including counseling, care, and opportunity (Hobfoll, 2009). Researchers consider social support as a kind of psychological assistance that can help people resist stress or psychological strain (Shumaker and Brownell, 1984). From the efforts to combat the pandemic, we can see that the timely help from the government, social organizations, and other people, being tangible support or intangible comfort, all contribute to the restoration of order during the pandemic. In light of this, the primary question to be addressed in this study is whether social assistance and support perceived or sensed by the public have the ability to enhance experienced PoM.

However, the global spread of the COVID-19 pandemic has made the restoration of normal order difficult. Whether the social mentality overwhelmed by the pandemic and following quarantine measures can be compensated by sensed long-term social support and remain healthy is also a major concern. Therefore, it is implied that time may play an important role in influencing social mentality. The present study seeks to build a cross-lagged model of social support and PoM, so as to verify if social support has a predictive effect on PoM. In other words, we examined the role of assistance from the government, society, and individuals in comforting the public under the long-term impact of the pandemic, responding to the call of “fighting against the virus instead of people.”

## Theories and Hypothesis

### Negative Social Emotions During the COVID-19 Epidemic

Peace of mind represents a moderately positive state, which some scholars have argued describes the sense of well-being from the Chinese cultural perspective (Lee et al., 2013). Chinese people favor moderate positive emotional experiences (Lu and Gilmour, 2004), as opposed to the high-arousal or hedonistic view of happiness more valued in the Western or individualist contexts (Okazaki, 2000; Oishi, 2001; Suh, 2002). Phrases, such as “harmony” and “balance,” are frequently found in the accounts of the conceptualization of happiness of the Chinese people (Tsai et al., 2006). Culture can shape the subjective meaning of happiness (Tsai et al., 2006). Tracing the ideological origins of Chinese culture reveals that PoM is a realm that Chinese people pursue and aspire to.

Taoism emphasizes the states of vacancy and stillness. In the Taoist classic Tao Te Ching, it is argued that “the state of vacancy should be brought to the utmost degree, and that of stillness guarded with unwearying vigor.” The Yellow Emperor's Classic of Internal Medicine mentions that “Yin is still and Yang is restless” and that “Yin remains inside to act as a guard for yang, while yang stays outside to serve as an actor of yin.” In addition, some Taoist classics also mention that a peaceful mind leads to wisdom, while a moving mind becomes faint (The Treatise on Sitting and Forgetting). The Seven Signatures of the Cloud Cage also indicated that “a quiet mind gives birth to a harmonious mind and a harmonious mind to a complete mind, whereas a restless mind gives birth to a swollen mind and a swollen mind to a wounded mind.”

Buddhism also attaches great importance to “stillness.” Buddhists pursue eliminating distracting thoughts and purifying the “six roots” through meditation. Buddhist meditation is also normally conducted in a quiet environment, which reinforces the peaceful inner world and is considered more conducive to the practice.

Confucianism, despite emphasizing “an initiation into the world,” also requires people to maintain a calm and peaceful mind. For example, Confucius said “The benevolent man is quiet” (Analects of Confucius, Yongye). Zhongyong or Doctrine of the Mean emphasizes the states of balance and harmony. The Great Learning mentions that “when you know where to stop, you have stability; when you have stability, you can be tranquil.” Zhu Xi, a Confucian scholar in the 12th century, believed that “There is a place of awareness in the state of stillness” (Zhuzi yulei). Some Confucian scholars, such as Wang Yangming, must sit quietly and meditate every day.

Other Chinese schools of thought also emphasize the pursuit of “calmness.” For example, Guan Zhong, a thinker and statesman during the Spring and Autumn Period (770–476 B.C.), believed that “If you clear your desire, your mind will be unblocked; if you are unblocked, you will be calm; if you are calm, you can concentrate on one thing. When the mind is concentrated, it is independent of all things, and when it is independent, it is clear about everything, and when it is clear about everything, it reaches the state of God” (Guanzi: Xinshu Shang). Zhuge Liang, a statesman during the Three Kingdoms period (189–280 A. D.), proposed in An Admonition to His Son that “This is a way of life for a man of virtue: to cultivate his character by keeping a peaceful mind, and nourish his morality by a frugal living.” Tao Qian, a poet and politician during the Six Dynasties period (220–589 A. D.), wrote “While picking asters 'neath the Eastern fence, my gaze upon the Southern mountain rests.” It, thus, appears that the well-being valued in Chinese culture is reflected in its pursuit of peace, stability, and permanence. Personal peace and tranquility are regarded as the highest realm that one can pursue (People's Forum “Special Planning Group”, 2014). Thus, the sense of well-being valued by people oriented to Chinese culture is vastly different from those oriented to western cultures. The infinite pursuit of PoM, since ancient times, has shaped the formation of the contours of the Chinese culture-specific affective well-being and served as an important indicator of the subjective well-being of people oriented to Chinese culture (Seligman, 2002).

Human beings are now facing huge uncertainty that is almost unprecedented—the unknown origin of the virus; the uncertain prospect for life, work, and economy; the uncertain international relations; and how the pandemic would progress. Under the circumstances, unstable negative emotions have become a prominent manifestation of public social psychology. Correspondingly, positive emotions, particularly the stable and restrained mental state of PoM, become less likely to be experienced.

Peace of mind refers to an emotional state that involves two components, namely, inner peace and internal harmony. Internal peace captures the states governed by low-arousal positive (LAP) affects, such as peacefulness, calmness, and serenity, which are felt when individuals are relieved of both the urge to evade negativeness and the desire to pursue positiveness (Lee et al., 2013). These emotions occur concomitantly and are more related to the actual correspondence to emergencies that happen from time to time in life (Corcoran et al., 2005; Milad et al., 2007). Internal harmony captures the state of harmony and balance, which refers to the process of self-control which individuals use to suppress intense feelings, such as anger, sorrow, and enthusiasm, and ensure that their emotional state fluctuates moderately (Shenkar and Ronen, 1987). The two aspects of PoM are intertwined such that individuals can either attain inner peace through the process of harmony or achieve harmony *via* maintaining a peaceful state of mind, thus constituting a coherent emotional state (Lee et al., 2013). The concept of PoM is used to measure the extent to which people are motivated to control themselves and cultivate positive emotional states, and this motivation may either be derived from their internal feelings or be evoked by external stimuli.

According to the self-determination theory, the basic needs of individuals include autonomy, competence, and relatedness (Deci and Ryan, 1985; Ryan and Deci, 2000). From the point of view of an individual, a sudden pandemic outbreak marked by a highly contagious virus and an alarming lethal rate has left the public incompetent to respond. Furthermore, the government-imposed regulations to contain the pandemic that people shall keep themselves in quarantine and practice social distancing have largely constrained the freedom or autonomy of the public. In other words, the spread of the virus and the following economic shutdown has restricted the disposable resources of the public. Besides, the social relations of the public were also restricted for the reason that they had to reduce physical contact. Therefore, it is difficult for the public to remain in a positive emotional state (Fernet et al., 2010), such as PoM, merely with their power for that, the basic needs of an individual were severely disrupted.

### As the Pandemic Rages, Social Support Continues to Play Its Role

What can evoke the peaceful mentality of the public? Previous research suggested that the unique benefit of PoM lies in its nature, which can be perceived as the continuous stable mindset that involves two affective characteristics, namely, “inner peace” and “internal harmony.” PoM emphasizes the emotional experiences of inner peace of individuals and their self-coordination and self-control process in achieving internal harmony (Lee et al., 2013). Existing studies on the antecedents of PoM have focused on either the emotional experiences that evoke inner peace (Xi, 2016) or human intervention and control (Xu, 2013; Xu et al., 2014). However, PoM is essentially a compound mental state that involves both inner peace and internal harmony (Lee et al., 2013). It requires both the natural arousal of emotions and the external intervention of human beings. On account of this, the current studies on PoM antecedents can be challenged for being biased in interpreting the causes behind PoM.

In contrast, social support has the ability to fill in the gap as it involves the stimulation of both natural arousal and human intervention (Hills and Argyle, 2002). Social support represents the “goodwill” conveyed in interpersonal interaction and serves as an important tool for employees to get on with their work and to maintain or replenish personal resources (Duffy et al., 2002; Halbesleben et al., 2014; Halbesleben and Wheeler, 2015). Besides, Hills and Argyle (2002) argued that the interpersonal relationship built on positive interaction gives rise to the generation of positive emotions, such as PoM. According to previous findings, harmonious interpersonal relationship is vital to individual well-being (Datu et al., 2018). In light of this, the present study proposes that social support has the ability to promote the PoM of individuals.

Clues can also be found in the response of the Chinese government to the pandemic. On January 25, 2020, the central committee of the Chinese government convened a meeting to establish a leading group for responding to the pandemic and make a comprehensive arrangement for pandemic prevention and control. After that, 31 provinces, autonomous regions, and municipalities have successively activated first-level public health emergency response, and all sectors of society have devoted all their efforts to pandemic prevention and control. Under this background, the public is becoming increasingly positive in mentality, an important manifestation of which has been the positive comments of the people on the efforts of local governments in pandemic prevention and control.

As discussed above, social support is claimed to positively affect PoM. First, the instrumental assistance involved in social support can help individuals to solve work-related problems and increase the possibility of completing their work, thereby alleviating their unhealthy mental state and promoting a more stable and positive mental state (Kim et al., 2020). What is more, social support, whether being instrumental or emotional assistance, represents the intimacy of human interaction, which is able to create a balanced state of mind for humans as social animals (Hayden et al., 2018). Thus, the emotional needs of individuals, such as a sense of belonging, can be met. Moreover, social support in diverse forms represents a variety of interpersonal interactions by which individuals lead an enriched life and obtain happiness. Finally, social support is a signal for benign interpersonal interaction. Benign interaction offers more outlets for channeling stress and negative emotions, so as to create a state of positiveness and happiness. Besides, research in evolutionary psychology has shown that interactions and alliances with others are an important psychological mechanism for humans to adapt to natural and social laws, which can help humans fend off attacks (Xu and Meng, 2004). To sum up, PoM can be nurtured and aroused by social support. Social support is an important antecedent of PoM.

### Aim of This Study

**Figure 1:**
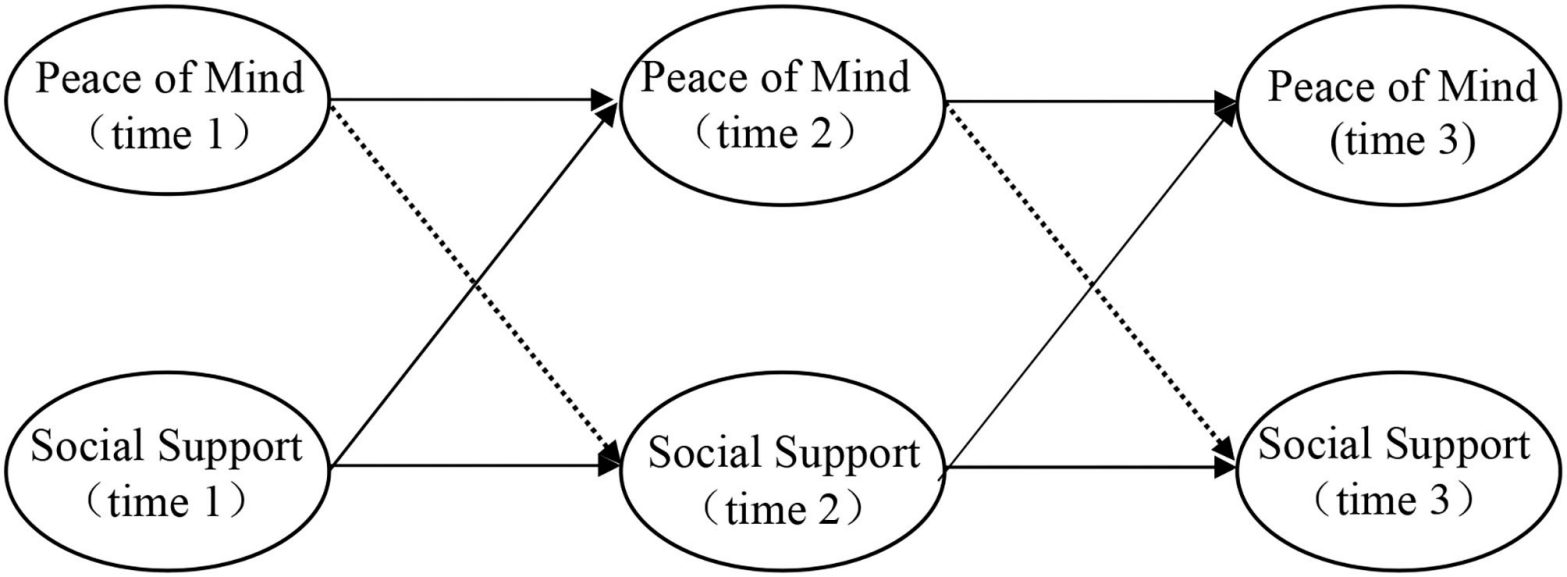
The hypothesized research model.

This study aims to investigate the time-cross relationship between PoM and social support in the times of the COVID-19 pandemic. The following research hypothesis is formulated: Social support has a significant time-lagged effect on PoM. The hypothesized research model is shown in Figure 1.

## Materials and Methods

### Participants and Data Collection

This study administered an online questionnaire survey among employees in seven different types of enterprises in Guangzhou, southeast of China, during the COVID-19 pandemic. To ensure that the time frame used in this survey conformed to our research design, we analyzed the existing literature and then decided on 1 month as the length of the time frame (Mitchell and James, 2001). We completed a three-phase data collection process and tried to obtain tracking data of the three times that all matched. The specific times for sampling were March 20, April 20, and May 20, 2020, all after the highest response level of public health emergency in Guangzhou was lifted and production was resumed with restrictions. In doing so, we set questions in the questionnaire which requested the participants to fill in their name initials and phone number as well as their relationship with the researchers. All questions were compulsory. The link of the electronic questionnaire survey was first sent to the human resource managers of the seven companies who forwarded it to their employees *via* email or WeChat, a Chinese social media. A total of 509 questionnaires were collected during the first phase of this study. The same steps were taken after 1 month and 2 months for the second and third phases of data collection, respectively. A total of 380 questionnaires were gathered for the second wave and 349 for the third. However, much to our regret that only 107 cases based on the three phases (with an effective rate of 20%) were finally selected after fine screening. Nonreactive analysis showed that participants who failed to participate in the follow-up survey did not differ significantly from those selected in the final sample in terms of the control variables, including age, gender, education level, and salary.

Of the participants, 48.59% were employed in foreign companies, 23.29% in private enterprises, 22.42% in public and government institutions, 2.8% in state-owned enterprises, and 2.79% in other types of organizations. With regard to the positions of participants in the organizations, front-line employees accounted for 44.86% of the total participants, grass-roots leaders for 28.97%, mid-level cadres for 24.29%, and senior supervisors for 1.86%. A majority of the participants held a college degree or above, which accounted for 86% of the total. The participants had an average age of 32 years (SD = 5.51). Of them, 52.34% were women.

### Research Instruments

#### Social Support

A simplified version of the Questionnaire on the Experience and Evaluation of Work (QEEW) was used to measure social support that reflected work resources (Van Veldhoven and Meijman, 1994). The scale was based on a 7-point Likert scale. Consistency analyses were performed with the three waves of data collected in this study, which showed that the consistency reliability coefficients were 0.87, 0.91, and 0.89, respectively. The total consistency coefficient of the three measurements was 0.91.

#### Peace of Mind

The 7-item PoM scale developed by Lee et al. (2013) was used to measure PoM, which was based on a 5-point Likert scale (1 = never, 5 = always). The total Cronbach's alpha coefficient in this sample was 0.90. The consistency reliability coefficients of the three waves of data were 0.76, 0.83, and 0.79, respectively.

#### Control Variables

Considering that the epidemic may have differential effects on different populations and enterprises (Aleman and Sommer, 2020; Broche-Perez et al., 2020; Brooks et al., 2020; Holmes et al., 2020; Liu et al., 2020; Rossi et al., 2020; Wang C. et al., 2020), we selected several demographic variables, including gender, marital status, education level, and position of employment, which distinguish the background of participants, and business ownership, which distinguishes business background, as control variables in this study.

## Results

### Descriptive and Correlation Analyses

Table 1 reports the means, variances, reliabilities, and correlation coefficients of the observed variables at each time point. The correlations between these two variables at the three time points had basically reached significance. Apart from the correlation between PoM-T3 and social support-T2, which reached the significance level of.05, all the correlations between these two variables at the three time points (PoM-T1, social support-T1; PoM-T2, social support-T2; PoM-T3, social support-T3) had reached a significance level of.01 or above. Besides, the correlations between PoM and social support at the same time point (i.e., PoM-T1 and social support-T1; PoM-T2 and social support-T2; PoM-T3 and social support-T3) all reached a significance level of.001.

**Table 1:** The means, variances, reliability coefficients, and correlation coefficients of the main variables.

**Variable**	**Mean**	**SD**	**1**	**2**	**3**	**4**	**5**	**6**	**7**	**8**	**9**	**10**	**11**
1. PoM-T1	3.49	0.66	0.78										
2. PoM-T2	3.38	0.62	0.40^***^	0.79									
3. PoM-T3	3.33	0.48	0.37^***^	0.43^***^	0.78								
4. Social Support-T1	3.55	0.73	0.55^***^	0.28^**^	0.28^**^	0.87							
5. Social Support-T2	3.27	0.87	0.35^***^	0.45^***^	0.22^*^	0.46^***^	0.91						
6. Social Support-T3	3.67	0.66	0.32^***^	0.25^**^	0.57^***^	0.53^***^	0.34^***^	0.89					
7. Gender	1.52	0.49	0.14	0.15	−0.04	0.09	0.14	−0.08	–				
8. Marriage	1.77	0.42	−0.09	0.10	0.12	−0.14	−0.00	−0.01	−0.00	–			
9. Education	3.01	0.47	−0.12	0.05	−0.00	0.00	−0.00	0.09	−0.00	−0.07	–		
10. Position	1.69	0.79	0.07	0.13	0.07	0.19	0.14	0.16	0.07	0.29	0.04	–	
11. Ownership	2.92	0.83	0.12	−0.03	−0.09	−0.04	0.05	−0.06	0.10	−0.09	0.02	−0.07	–

*N = 107*;

*** *P < 0.001*,

** *P < 0.01*,

* *P < 0.05; Consistency coefficients are on the diagonal*.

### Model Test and Confirmatory Factor Analysis

Prior to validating the hypotheses, we tested a measurement model which included all the six latent variables (i.e., PoM and social support measured at the three points) as the baseline model (refer to the two-step method; Anderson and Gerbing, 1988). The goodness-of-fit index was satisfactory [χ^2^(282) = 418.918, *p* < 0.001, Comparative Fit Index (CFI) = 0.926, Tucker Lewis Index (TLI) = 0.915, Root Mean Square Error of Approximation (RMSEA) = 0.068].

Then, we entered PoM and social support at each point of time into the same model to test their discriminant validity and convergent validity at the same time (M3: PoMT1, Social supportT1; M4: PoMT2, Social supportT2; M5: PoMT3, Social supportT3). The results of confirmatory factor analysis showed that the two variables were independent from each other at all the three points. As shown in Table 2, the two-factor model had a good fit at T1 [χ^2^(19) = 25.188, *p* > 0.05, CFI = 0.987, TLI = 0.981, RMSEA = 0.055], which was superior to the fit reported for the one-factor model at T1. Similar results for the two-factor model were presented at T2 [χ^2^(26) = 34.378, *p* > 0.05, CFI = 0.987, TLI = 0.982, RMSEA = 0.055] and T3 [χ^2^(26) = 30.116, *p* > 0.05, CFI = 0.991, TLI = 0.987, RMSEA = 0.038], which were better than the fit indexes reported for the one-factor model at T2 and T3 points, respectively.

**Table 2:** Confirmatory factor analysis.

**Model**	**M1. Hypothesized model**	**M2. Baseline model**	**M3. T1-Two-factor model**	**M4. T2-Two-factor model**	**M5. T3-Two-factor model**
Factors	Model with paths of the cross-lag effects between 6 factors at 3 points	T1-, T2-, T3-Social support and T1-, T2-, T3-PoM	T1-Social support and T1-PoM	T2-Social support and T2-PoM	T3-Social support and T3-PoM
χ^2^	407.368^***^	418.918^***^	25.188	34.378	30.116
df	279	282	19	26	26
CFI	0.931	0.926	0.987	0.987	0.991
TLI	0.919	0.915	0.981	0.982	0.987
RMSEA	0.066	0.068	0.055	0.055	0.038
Confidence Interval of RMSEA	[0.052, 0.079]	[0.054, 0.081]	[0.000, 0.108]	[0.000, 0.100]	[0.000, 0.089]
SRMR	0.066	0.081	0.050	0.044	0.047
PClose	0.036	0.021	0.401	0.403	0.594

*N = 107;*

*** *P < 0.001*.

Comparing the three time-independent two-factor models (M3, M4, M5) to the baseline model (M2, the measurement model with all the six latent variables), we found that the fit indexes had no significant change (comparison of M3 and M2 in Table 2: Δχ^2^/Δdf = 1.50, *p* > 0.05; comparison of M4 and M2 in Table 2: Δχ^2^/Δdf = 1.50, *p* > 0.05; comparison of M5 and M2 in Table 2: Δχ^2^/Δdf = 1.52, *p* > 0.05), which confirmed that the baseline model has a good discriminant validity and convergent validity.

Finally, we tested the hypothesized model of the cross-lag effect in this study, which showed a good fit [M1: χ^2^(279) = 407.368, *p* < 0.001, CFI = 0.931, TLI = 0.919, RMSEA = 0.066]. In addition, we compared the fit indexes of the hypothesized model (M1) and the baseline model (M2), which showed no significant change between them (Δχ^2^/Δdf = 3.85, *p* < 0.05). It, thus, proved that the hypothesized cross-lag model (M1) in this study is more acceptable, providing support for the hypothesis testing.

### Methodological Aspect of Structural Equation Modeling for Time-Lagged Data

The cross-lag model comes from time-series analysis, is estimated after detrending the time-series data for each employee. Detrending attempts to remove systematic changes in the scores over time to prevent spurious associations due to the variables changing over time (Grimm et al., 2021). The cross-lag model is often specified in the structural equation modeling framework following Joreskog (1979) because of the multiple outcome variables of the model (i.e., *PoM*_*ti*_ and *Social support*_*ti*_) and applied to raw data (data that has not been detrended). The cross-lag model for the longitudinal PoM and social supporting data is written as


(1) $$\begin{array}{l} PoM_{ti}=\beta_{00t}+\beta_{10t}\cdot Social\;support_{t-1i}\\ \;+\beta_{20t}\cdot PoM_{t-1i}+u_{sti} \end{array}$$

(2) $$\begin{array}{l} Social\;support_{ti}=\beta_{01t}+\beta_{11t}\cdot PoM_{t-1i}\\ \;+\beta_{21t}\cdot Social\;support_{t-1i}+u_{pti} \end{array}$$

where *PoM*_*ti*_ and *Social support*_*ti*_ are the PoM and social support degrees for employee *i* in time *t* (e.g., time 3, 2020.05.20), respectively, and *PoM*_*t*−1*i*_ and *Social support*_*t*−1*i*_ are the PoM and social support degrees for employee *i* in time *t*−1 (e.g., time 2, 2020.04.20). The parameters of the cross-lag model include β_00*t*_ and β_01*t*_, which are time-dependent intercepts and predicted PoM and social support degrees in time *t* when PoM and social support degrees are zero at the previous time; β_10*t*_ and β_11*t*_, which are time-dependent autoregressive parameters representing the consistency of the between-person differences in PoM and social support, respectively; β_20*t*_ and β_21*t*_, which are time-dependent cross-lag parameters and often the parameters of most interest because they represent the effect of prior social support on PoM controlling for prior PoM and the effect of prior PoM on social support controlling for prior social support, respectively; and *u*_*sti*_ and *u*_*pti*_, which are the time-dependent residuals and part of *PoM*_*ti*_ and *Social support*_*ti*_, respectively, that are linearly unrelated to *PoM*_*t*−1*i*_ and *Social support*_*t*−1*i*_. The *p* and *s* in *u*_*sti*_ and *u*_*pti*_ are to denote the residual PoM and social support, respectively. The variances of *u*_*sti*_ and *u*_*pti*_ are estimated as well as their covariance. Additionally, the initial scores for PoM and social support (i.e., time 1 scores for our illustrative data) are allowed to covary. The autoregressive and cross-lag effects can be specified to be independent of time (e.g., β_10*t*_ = β_10_), and constraining them to be equal over time can increase statistical power, but damage model fit.

### Hypothesis Test Using Cross-Lag Model

We tested the hypothesis using a structural equations model. A cross-lagged latent factor model was used to test the research hypothesis, i.e., the time-lagged effects of PoM on social support and of social support on PoM. To ensure an appropriate sample size to parameter estimate ratio, we used the scale means to indicate each latent factor of PoM and social support across the three time points.

Although the causation from covariance structure cannot be proved alone, cross-lagged regression analysis (Kenny and Harackiewicz, 1979) provided more conclusive evidence of causal precedence among social support and PoM variables than the cross-sectional relationships that have been reported to date. We first tested the goodness of fit of the hypothesized model (as shown in Table 2), which fit the data quite well: [M1: χ^2^(279) = 407.368, *p* < 0.001, CFI = 0.931, TLI = 0.919, RMSEA = 0.066].

Then, we estimated a model that set the path between these two variables when controlling the effects of control variables {χ^2^(380) = 540.918, *p* < 0.001, CFI = 0.915, TLI = 0.898, RMSEA = 0.063 [0.051, 0.075], Pclose = 0.043, SRMR = 0.067}. Its goodness of fit did show a significant difference from the hypothesized model (M1) (Δχ^2^/Δdf = 1.32, *p* > 0.05). That is, both models showed a good structure.

**Figure 2:**
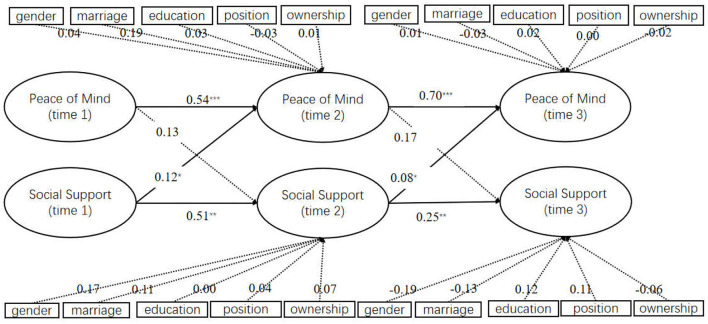
Cross-lag model of social support and PoM. ***P < 0.001, ^**^P < 0.01, ^*^P < 0.05. χ^2^(380) = 540.918, *p* < 0.001, CFI = 0.915, TLI = 0.898, RMSEA = 0.063 [0.051, 0.075], Pclose = 0.043, SRMR = 0.067.

The results (as shown in Figure 2) showed that as we expected, social support-T1 had a significant predictive effect on PoM-T2 (β = 0.16, SE = 0.09, *p* < 0.05) and social support-T2 had a significant predictive effect on PoM-T3 (β = 0.38, SE = 0.19, *p* < 0.05). This indicated that social support had a cross-lag effect on PoM and this effect had time-span stability and long-term characteristic. However, the cross-lag effect of PoM on social support turns out to be insignificant, including the predictive effect of PoM-T1 on social support-T2 (β = 0.04, SE = 0.07, *p* > 0.05) and the predictive effect of PoM-T2 on social support-T3 (β = 0.13, SE = 0.09, *p* > 0.05). Thus, the research hypothesis was verified.

**Figure 3:**
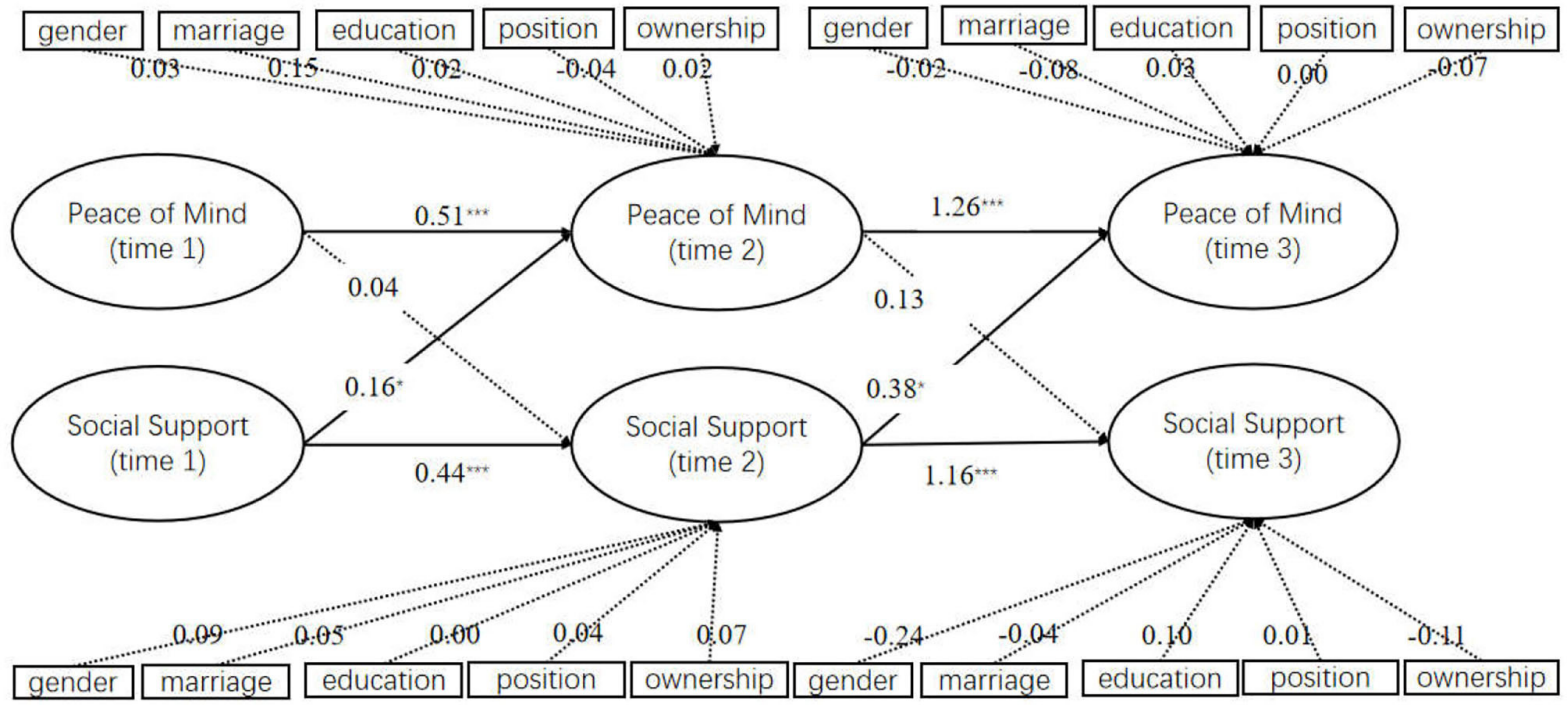
Cross-lag model of social support and PoM *via* bootstrapping. ^***^P < 0.001, ^*^P < 0.05.

Finally, to ensure the robustness of the findings, this study further used structural equation modeling combined with a Bootstrap algorithm to verify the cross-lagged relationship between PoM and social support. After 1,000 times of sampling using the bias-corrected bootstrap method, the results remained consistent with the previous findings with only minor variations on the path coefficients: as shown in Figure 3, social support-T1 had a significant predictive effect on PoM-T2 (β = 0.12, SE = 0.08, *p* < 0.05); social support-T2 had a significant predictive effect on PoM-T3 (β = 0.08, SE = 0.04, *p* < 0.05). It was, thus, confirmed that social support had a cross-lag effect on PoM, corroborating the research hypothesis.

Then, we estimated a model that set the path between these two variables when controlling the effects of control variables {χ^2^(380) = 540.918, *p* < 0.001, CFI = 0.915, TLI = 0.898, RMSEA = 0.063 [0.051, 0.075], Pclose = 0.043, SRMR = 0.067}. Its goodness of fit did show a significant difference from the hypothesized model (M1) (Δχ^2^/Δdf = 1.32, *p* > 0.05). That is, both models showed a good structure.

The results (as shown in Figure 2) showed that as we expected, social support-T1 had a significant predictive effect on PoM-T2 (β = 0.16, SE = 0.09, *p* < 0.05) and social support-T2 had a significant predictive effect on PoM-T3 (β = 0.38, SE = 0.19, *p* < 0.05). This indicated that social support had a cross-lag effect on PoM and this effect had time-span stability and long-term characteristic. However, the cross-lag effect of PoM on social support turns out to be insignificant, including the predictive effect of PoM-T1 on social support-T2 (β = 0.04, SE = 0.07, *p* > 0.05) and the predictive effect of PoM-T2 on social support-T3 (β = 0.13, SE = 0.09, *p* > 0.05). Thus, the research hypothesis was verified.

Finally, to ensure the robustness of the findings, this study further used structural equation modeling combined with a Bootstrap algorithm to verify the cross-lagged relationship between PoM and social support. After 1,000 times of sampling using the bias-corrected bootstrap method, the results remained consistent with the previous findings with only minor variations on the path coefficients: as shown in Figure 3, social support-T1 had a significant predictive effect on PoM-T2 (β = 0.12, SE = 0.08, *p* <0.05); social support-T2 had a significant predictive effect on PoM-T3 (β = 0.08, SE = 0.04, *p* <0.05). It was, thus, confirmed that social support had a cross-lag effect on PoM, corroborating the research hypothesis.

## Discussion

The empirical evidence presented in this study supported the research hypothesis. Specifically, social support had a cross-lag effect on PoM, whereas PoM did not have a cross-lag effect on social support. This corroborates previous studies on the relationship between social support and positive emotions (Shumaker and Brownell, 1984; Sun et al., 2011). However, our study took it a step further by validating the causal relationship between the two variables through cross-lag analysis, rather than just correlation between them. We, thus, propose that in a public health emergency like the COVID-19 pandemic, social support and external assistance, as well as a positive interpersonal interaction experience, are vital to the cultivation of the PoM of individuals and improvement of perceived well-being at the workplace.

### Theoretical Implications

First, this study filled one of the gaps in the existing research which has predominantly addressed the adaptive role of positive emotions within the framework of Fredrickson's (2001) the broaden-and-build theory of (Anjum et al., 2014; Ariyabuddhiphongs and Pratchawittayagorn, 2014; Datu, 2017; Liang et al., 2020). These studies only emphasized the “positivity” of PoM and considered it the same as other activating positive emotions aroused by personal positive experiences (Fredrickson, 2001). For example, PoM can be aroused by mindfulness training where individuals are cultivated to recall or imagine being in a comfortable and relaxing environment multiple times (Yu et al., 2019). Such approaches, however, fell short of highlighting PoM as a low-arousal, stable, and restrained emotional state (Lee et al., 2013). This study, from the perspective of interpersonal interaction, revealed that the positive state of PoM arises not only from personal momentary positive experiences (Yu et al., 2019) but also from the support of others on a long-term basis, which further leads to positive interpersonal interaction. This study contributed to the broadening of the scope of positive psychology.

Second, this study adopted a cross-lag approach to testing the reciprocal relationship between PoM and social support that has rarely been touched upon in previous research (Yang et al., 2018; Xi et al., 2020). In this regard, a stable causal relationship between social support and PoM was identified, which provided more insights into the research on the antecedents of the PoM. More specifically, this study, on the one hand, examined a potential reciprocal relationship between PoM and social support with empirical evidence. On the other hand, the research findings confirmed a one-way causal relationship between social support and PoM, which identified an important antecedent of PoM. Previous studies have dealt with PoM antecedents from the dimension of natural arousal or human intervention. However, this study confirmed that social support has a significant impact on PoM in both ways and provided a holistic understanding of the paths of PoM arousal.

Third, this study explored the effective ways to help people restore peaceful and balanced states in the time of public health emergencies. On the one hand, we identified social support as an important means of (re-)building positive social public mentality; on the other hand, we found that PoM-T1 has a significant effect on PoM-T2. Therefore, the interactive effect of time and social support enables individuals to achieve and maintain their state of PoM, i.e., subjective well-being. The effect of time has not drawn sufficient attention in previous studies (Shumaker and Brownell, 1984; Sun et al., 2011). We investigated the effects of the two important factors, social support and time, on PoM, which revealed that the state of PoM grows with the increase of social support and over time. Therefore, in a public health emergency, the provision of social support should be utilized along with rapid response and can thus help people restore mentality.

### Practical Implications

The research findings are of great significance for the implementation of psychological assistance during the pandemic. Considering that the social mentality of the public is dynamic and complicated, it is necessary to adapt the intervention measures to the given context. In fact, most people are capable of adjusting their own affective states, and the increase in social support can help make the current situation even better, for that it meets the basic needs of the public through stabilizing their moods and helping them get rid of negative feelings. In addition, some people who are greatly affected by the pandemic and suffer from an anxious state of mind can also have their unhealthy mental state suppressed and ease out anxious feelings by obtaining targeted external support. This study has not only provided an explanation for the negative mental state of the people during the pandemic quarantine, but it also provided applicable technical information on psychological assistance for the public during public health emergencies.

### Limitations and Prospects

Although credits should be given to this study for its theoretical and practical contributions, there are also limitations that need to be addressed in future studies. First, although the time frame used in this study ensured the validity of the causal explanation and minimized the interference of common method bias, the samples suffered from severe data loss due to poor data matching between the three rounds of the longitudinal study. Future studies are advised to use more rigorous methods in the longitudinal tracking investigation of relevant models. Second, the study used merely a cross-lag research design, which may not be enough to test the long-term stability of PoM. Diversified research designs are expected in future research. Third, this study showed that both PoM and social support increased over the three points in time, which could be contributed to social contextual factors during the pandemic, such as the restoration of social order, reduction of social distancing, or increased public trust in the government. Future studies are needed to examine the causes behind them. Finally, the states of mind or feelings of participants affected by the pandemic may be distinctive, which may make these findings less generalizable. Further studies with more focus on the effect of context are therefore suggested.

## Data Availability Statement

The raw data supporting the conclusions of this article will be made available by the authors, without undue reservation.

## Ethics Statement

The studies involving human participants were reviewed and approved by Academic Ethics Committee of Nanjing University of Aeronautics and Astronautics. The patients/participants provided their written informed consent to participate in this study.

## Author Contributions

YX involved in conceptualization, methodology, writing—original draft preparation, and formal analysis. LZ involved in the investigation, validation, and visualization. YW involved in conceptualization, review and editing, and project administration. All authors contributed to the article and approved the submitted version.

## Conflict of Interest

The authors declare that the research was conducted in the absence of any commercial or financial relationships that could be construed as a potential conflict of interest.

## Publisher's Note

All claims expressed in this article are solely those of the authors and do not necessarily represent those of their affiliated organizations, or those of the publisher, the editors and the reviewers. Any product that may be evaluated in this article, or claim that may be made by its manufacturer, is not guaranteed or endorsed by the publisher.
